# P005. Efficacy Type-A Botulinum toxin treatment in a multidisciplinary setting for chronic headache

**DOI:** 10.1186/1129-2377-16-S1-A185

**Published:** 2015-09-28

**Authors:** Giuseppe Vescio, Amerigo Costa, Aida Squillace, Rosario Iannacchero

**Affiliations:** Department of Health Science, Magna Graecia University, Catanzaro, Italy; Centre for Headache and Adaptive Disorders, Unit of Neurology, Department of Neuroscience and Sense Organs, Azienda Ospedaliera “Pugliese-Ciaccio”, Catanzaro, Italy

## Background

Type-A botulinum toxin (BoNTA) therapy has emerged as an effective treatment for chronic headache (CH) [1]. No conclusive data exists about BoNTA efficacy in CH comorbid with anxiety and depression disorders. Our open prospective study aimed to evaluate CH BoNTA treatment efficacy regarding clinical and psychopathological variables in a multidisciplinary setting.

## Materials and methods

We treated 32 CH patients (8 males; 24 females; 44.76±11.23 mean age) with 190-units BoNTA injections. Sessions took place from January 2014 to June 2015 once every 3 months; patients received headache education; at the baseline (T0) and at the final follow-up (T1) patients completed a headache diary, pain Numerical Rating Scale (NRS), Migraine Disability Assessment (MIDAS), Zung Self-Rating Anxiety Scale (SAS) and Zung Self-Rating Depression Scale (SDS). We considered patients responders if they had > 50% reduction in headache frequency and/or pain intensity compared with baseline. Using SOFA Statistics 1.4.4 software, we calculated descriptive indicators and evaluated treatment effect using paired-samples Student's *t*-test on clinical and psychosocial variables between T0 and T1. We set p < 0.05 as threshold of statistical significance.

## Results

At T0 headache frequency (M±DS) was 23.71±5.38 headache days/month; NRS score was 9.10±0.94; MIDAS score was 57.59±24.72; SAS score was 48.38±12.16; SDS score was 46.68±14.31. At T1 headache frequency (M±DS) was 9.14±8.31 headache days/month; NRS score was 6.33±2.33; MIDAS score was 21.517±20.19; SAS score was 39.38±9.19; SDS score was 36.01±10.32. Twenty-one patients (65.25%) showed psychopathological comorbidity. We observed response to treatment in 23 patients (71.87%) with a statistically significant treatment effect on headache frequency (*t* = 8.539; p < 0.001), pain intensity (*t* = 5.451; p < 0.001), disability (*t* = 5.701; p < 0.001), anxiety (*t* = 3.457; p < 0.005) and depression (*t* = 2.59; p < 0.05).

## Conclusions

Our data support that BoNTA in a multidisciplinary setting is an effective treatment for chronic headache, potentially addressing pain-related affective disorders. Further developments of our study could evaluate the effectiveness of enhanced psychological interventions, such as stress management, as a complement to the biomedical treatment.

Written informed consent to publish was obtained from the patient(s).
